# Use of recombinant porcine β-defensin 2 as a medicated feed additive for weaned piglets

**DOI:** 10.1038/srep26790

**Published:** 2016-05-26

**Authors:** Zixin Peng, Anru Wang, Linqi Xie, Weiping Song, Jie Wang, Zhe Yin, Dongsheng Zhou, Fengqin Li

**Affiliations:** 1 Microbiology Laboratory, China National Center for Food Safety Risk Assessment, No. 7 Panjiayuannanli Road, Chaoyang District, Beijing, 100021, China; 2 State Key Laboratory of Direct-Fed Microbial Engineering, No. B-3 Northern Territory of Zhongguancun Dongsheng Science and Technology Park, Haidian District, Beijing, 100193, China; 3 State Key Laboratory of Pathogen and Biosecurity, Beijing Institute of Microbiology and Epidemiology, No. 15 Fengtaidongdajie Street, Fengtai District, Beijing, 100071, China

## Abstract

Post-weaning diarrhoea (PWD) in piglets is associated with colonization of the intestine with bacterial pathogens. In this study, we evaluated the use of recombinant porcine β-defensin 2 (rpBD2) as a medicated feed additive for weaned piglets. The crude extract from the culture supernatant of rpBD2-expressing *Pichia pastoris* was used as a medicated feed additive for weaned piglets. Dietary treatments included a positive control (basal diet + antibiotics, designated PC) and three different rpBD2 treatments without antibiotics (basal diet supplemented with 1, 5, or 15 g of crude rpBD2/kg basal diet, designated 1PD, 5PD, and 15PD, respectively). Of all the treatments, 5PD had the greatest impact on the weaned piglets. It increased their body weight, average daily weight gain, average daily feed intake, and intestinal villus height in the duodenum and jejunum, and reduced the incidence of PWD. The diversity of the cecal digesta and mucosa microflora was compared between the weaned piglets in the PC and 5PD groups. Piglets treated with 5PD had lower diversity indices and fewer bacterial pathogens in their cecal digesta and mucosa than the PC group. Our results demonstrate that crude rpBD2 could provide an alternative to the traditional antibiotic feed additives given to weaned piglets.

Post-weaning diarrhoea (PWD) is caused by a number of bacterial pathogens, including *Escherichia coli*, *Clostridium* spp., and *Lawsonia* spp., and is a significant gastrointestinal disease in pigs, entailing high economic losses in pig herds^1^,^2^,^3^. For decades, antibiotic growth promoters have been widely used in piglets to reduce enteric infections and improve the composition of the intestinal microflora, thereby reducing the risk of PWD^4^,^5^. The limitations of dietary antibiotics include the presence of drug residues in edible animal products and the reduction of the innate immune defences of young animals^6^,^7^. Therefore, natural products are urgently required for use as alternatives to antibiotic feed additives^8^,^9^,^10^,^11^,^12^.

Defensins are a family of endogenous cationic antimicrobial peptides that play an important role in the innate and adaptive immune systems of mammals, and provide protection against intestinal bacterial infections by modulating the composition of the intestinal microbiota^13^,^14^. Bacteria are less able to develop resistance to defensins than to traditional antibiotics, because defensins disrupt the bacterial membrane by forming non-specific electrostatic interactions with the membrane lipid components^15^. Therefore, the administration of defensins is a potentially novel therapeutic strategy for inflammatory and infectious diseases of the gastrointestinal tract^16^, and may present a promising alternative to the traditional antibiotic feed additives used in the livestock industry.

Mature porcine β-defensin 2 (pBD2), whose expression in the pig intestine is induced by infection with intestinal pathogens^14^, exerts strong antimicrobial activity against a broad range of pathogenic intestinal bacteria, with very limited haemolytic activity against porcine red blood cells^17^. With a triple-stranded β-sheet fold and a framework of six disulfide-linked cysteines, the arginine-rich cationic peptide exerts its antimicrobial and cytotoxic effects by permeabilizing the target membrane when it inserts into it in response to the electrical forces that act on the positively charged defensin molecule^17^,^18^. As shown in our previous study^19^, recombinant pBD2 overexpressed in *Pichia pastoris* can pass through the stomach and intestine and tolerate feed pellet processing with no loss of its antimicrobial activity because its thermal and pH stability and proteolytic resistance are high. Furthermore, a crude extract of the culture supernatant of rpBD2-expressing *P. pastoris* (designated ‘crude rpBD2’) can be added directly to feed without rpBD2 purification. This advantage reduces the production costs and allows its application to be scaled up. The objective of this follow-up study was to rigorously evaluate the effects of dietary supplementation with crude rpBD2 as a medicated feed additive on the growth performance, intestinal morphology, and intestinal microflora of weaned piglets on a commercial farm.

## Results

### Experimental design

A clear rpBD2 band was observed following tricine–sodium dodecyl sulfate–polyacrylamide gel electrophoresis (tricine–SDS–PAGE) of the crude rpBD2 powder (concentration 474.8 ± 34.2 mg/g). The application of crude rpBD2 caused an obvious inhibition zone on a bacterial lawn of *Staphylococcus aureus* ATCC 6538. The minimal inhibitory concentrations of crude rpBD2 against a broad range of pig pathogenic bacteria were previously shown to range from 32 to 128 μg/mL^19^. Three different rpBD2-supplemented feeds without antibiotics (basal diet supplemented with 1, 5, or 15 g crude rpBD2/kg, designated 1PD, 5PD, and 15PD, respectively) and the positive control (PC; basal diet + 1.5% crude *P. pastoris* X-33 + 200 mg/kg 10% colistin sulfate + 1,000 mg/kg 10% zinc bacitracin) were given to weaned piglets on a commercial farm to determine their effects on the growth performance, incidence of PWD, small intestinal morphology, and intestinal microflora of the piglets. Our aim was to identify the appropriate dose of the crude rpBD2 additive and confirm its utility as an alternative to traditional antibiotic feed additives.

### Growth performance and incidence of PWD

The growth performance and incidence of PWD in all the weaned piglet groups tested are listed in Table 1. In phase I, the average daily weight gain (ADG) and average daily feed intake (ADFI) values of the 5PD group were significantly higher than those of the 1PD and 15PD groups, but were not significantly different from those of the PC group. In phase II, the ADG and ADFI values of the 5PD group were superior to those of the 1PD, 15PD, and PC groups. Throughout the experimental period, the overall values for body weight (BW), ADG, and ADFI were significantly higher and the incidence of PWD was significantly lower in the 5PD group than in the 1PD, 15PD, and PC groups, whereas the 5PD and PC groups had similar feed conversion (G/F) values (1.80 vs 1.81, respectively). Therefore, in general, 5PD was more effective than 1PD, 15PD, or PC in improving the growth performance of weaned piglets and reducing the incidence of PWD.

### Small intestinal morphology

As shown in Table 2, the villus height values in the duodenum and jejunum were significantly greater in the 5PD group than in the 1PD, 15PD, or PC group. Dietary treatment did not appear to affect the crypt depth in the duodenum in any of the PD treatment groups, but the duodenal crypt depth was much shallower in the 5PD group than in the PC group (317.6 μm vs 351.7 μm, respectively). The crypt depth in the ileum was significantly shallower in the 5PD group than in the 1PD, 15PD, or PC group. No significant differences in the villus height/crypt depth ratio were observed among the treatment groups. Overall, 5PD was more effective than 1PD, 15PD, or PC in improving the small intestinal morphology of the weaned piglets. Representative micrographs of small intestinal morphology of the PC and 5PD groups are shown in Fig. S1.

### Intestinal bacterial community

**Figure 1:**
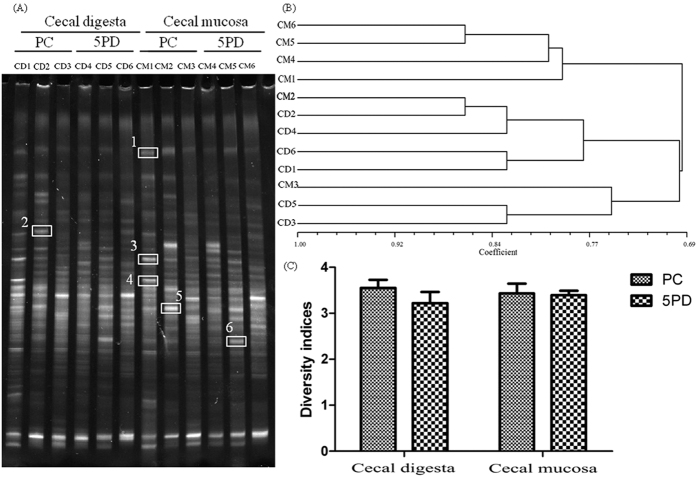
Denaturing gradient gel electrophoresis (DGGE) results. **(A) DGGE profiles.** 12 DGGE profiles of the PCR products from the V3 region of the 16S rRNA gene from the PC and 5PD cecal digesta and mucosal samples on day 28. Bands identified from the 16S rRNA gene clone libraries are numbered and indicated with square frames. The bands are also described in Table 3. (**B**) **Clustered DGGE profiles.** UPGMA clustering diagram of Dice similarity indices of the 12 DGGE profiles. **(C) Calculated diversity indices.** Diversity indices were used to compare the PC and 5PD cecal digesta and mucosa samples on day 28. Triplicate observations from three individual piglets were made and data are means ± standard errors (SE).

In total, 12 denaturing gradient gel electrophoresis (DGGE) profiles were generated when DGGE was used to analyse the cecal digesta and mucosal samples of three piglets randomly selected from the PC and 5PD pens on day 28 (Fig. 1A). The relative intensities of bands 1, 2, 3, 4, and 5 were considerably lower in the 5PD group than in the PC group and, moreover, the bands 1, 2, and 4 in the 5PD group were below the limit of detection. By contrast, the relative intensity of band 6 was higher in the 5PD group than in the PC group. Band 2 was present in the cecal digesta but not found in the mucosal samples (CD2 vs CM2 profile in Fig. 1A, respectively), whereas band 5 was constantly visible in the cecal mucosal samples but almost invisible in the digesta samples.

The 16S rRNA-DGGE bands had sequence similarities to those of organisms closely related to *Solobacterium moorei* NR_113039.1 (band 1), *Helicobacter canadensis* NR_115104.1 (band 2), *Eubacterium eligens* NR_074613.1 (band 3), *Coprococcus comes* NR_044048.1 (band 4), *Clostridium polysaccharolyticum* NR_119085.1 (band 5), and *Fusicatenibacter saccharivorans* NR_114326.1 (band 6), with similarities of 87%, 100%, 100%, 100%, 99%, and 100%, respectively (Table 3).

The 12 DGGE profiles formed a coherent cluster, with similarity indices above 69% (Fig. 1B). The cecal mucosal profiles of the 5PD group (CM4/CM5/CM6) had the highest intra-group similarity (>80%) when compared with the other three groups (~69% each): cecal digesta PC group (CD1/CD2/CD3), cecal digesta 5PD group (CD4/CD5/CD6), and cecal mucosa PC group (CM1/CM2/CM3). The two DGGE profiles CM2 and CD2 had the highest pairwise similarity (>86%) compared with all the other pairwise similarity values, which ranged from 69% to 75%. These results indicate that rpBD2 increased the coherence of the mucosal microbiota in the piglet caecum, thereby reducing the diversity of microflora.

The Shannon index of diversity (*H*′) for each cecal digesta and mucosa sample was lower in the 5PD group than in the PC group, but no statistical significant difference was observed among the four groups. Furthermore, in the PC and 5PD groups, the Shannon indices were more similar among the cecal mucosal samples than among the digesta samples (Fig. 1C). These results indicate that rpBD2 reduces the intestinal pathogenic microbiota in the piglet cecal digesta more than that in the mucosa.

## Discussion

Defensin is an important component of the nonspecific immune system and acts as a gene-encoded antibiotic to repel the assault of diverse infectious agents, including bacteria, viruses, fungi, and parasites^18^,^20^,^21^,^22^. In this study, we have shown that dietary supplementation with crude rpBD2 improved the growth performance of weaned piglets and reduced the incidence of PWD more effectively than antibiotic supplementation. These results suggest that rpBD2 can be used as a growth promoter and medicinal agent instead of antibiotics to improve the growth performance of piglets and prevent PWD. Importantly, 5 g/kg crude rpBD2 most effectively improved the growth performance of the weaned piglets and reduced the incidence of PWD. Lower (1 g/kg) or higher (15 g/kg) doses of rpBD2 were less effective. Why higher dose of rpBD2 did not acquire better growth performance is unclear. Probably, higher dose of rpBD2 would inhibit the survival of probiotic in porcine intestines as shown in our previous study^19^. Notably, significant improvement in piglet growth performance was observed in phase II but not in phase I, probably because piglet growth in response to rpBD2 occurred at least 2 weeks after weaning.

The intestinal morphology is indicative of pig gut health. Villus height and the villus height/crypt depth ratio correlate with epithelial turnover, and longer villi are associated with active cell mitosis and greater nutrient absorption^23^,^24^. After weaning, the villus height is reduced and the crypt depth increases, primarily because feed intake decreases immediately after weaning^23^. In swine and broiler models, dietary supplementation with probiotics, antimicrobial peptides (cecropin AD, antimicrobial peptide A3, or antimicrobial peptide P5), or glucose has a beneficial effect on mucosal development, increasing the villus height and causing weight gain^25^,^26^,^27^,^28^,^29^,^30^. Shorter villi and deeper crypts are associated with the presence of toxins or pathogens^31^. In this study, greater villus heights and shallower crypt depths were observed in the weaned piglets treated with 5PD compared with the PC group, and this may have contributed to their improved growth performance.

The pig large intestine is densely colonized with bacteria, but little is known about the microfloral composition and its variation after weaning, which significantly affect piglet growth performance and health^3^,^32^. During weaning, major quantitative and qualitative changes occur in the composition of the piglet intestinal microbiota, providing an opportunity for pathogenic coliforms and other bacteria to invade, contributing to gastric disorders and hence reduced performance^33^. Dietary supplementation with an antimicrobial peptide, such as a fusion peptide of lactoferricin and lactoferrampin, potato protein, antimicrobial peptide A3, cecropin AD, or antimicrobial peptide P5, reduces the total numbers of aerobes while simultaneously enhancing the total amount of anaerobes and beneficial lactobacilli in the intestines of weaned piglets^24^,^25^,^27^,^28^,^33^.

The intestinal microbial community of pigs has been investigated in several studies with traditional microbial culture techniques. However, many strictly anaerobic intestinal bacteria are difficult to culture and remain undetectable with conventional techniques. The combined use of 16S rRNA gene-targeted PCR, DGGE, cloning, and sequencing is a powerful approach to identifying the gastrointestinal microbiota, including bacteria that are difficult to culture^34^,^35^. As shown with PCR–DGGE, rpBD2 potentially suppresses harmful intestinal microflora in weaning piglets. The five DGGE bands attenuated in the 5PD-treated group relative to the PC group were from organisms most similar to *S. moorei*, *H. Canadensis*, *E. eligens*, *C. comes*, and *C. polysaccharolyticum*. Another band, representing *F. saccharivorans*, was more intense in the 5PD-treated group than in the PC group. *S. moorei* is a non-spore-forming, strictly anaerobic Gram-positive bacillus that has been identified in specimens from human patients with dental diseases and wound infections^36^. Because the partial 16S rRNA sequences showed a similarity to *S. moorei* much lower than 97% in this study, the corresponding bacterium probably represents a new *Solobacterium* species. *H. Canadensis* has been isolated from swine faeces in Europe and has been associated with diarrhoea^37^. The administration of clindamycin increases the bacterial load of *E. eligens* in standardized human faecal microbiota^38^. *C. comes* is an anaerobic Gram-positive coccoid rod isolated from the faecal flora and sera of patients with Crohn’s disease^39^. *C. polysaccharolyticum* is a common bacterium in animal intestines^40^, and *Clostridium* species are common anaerobic pathogens in the pig intestines^28^,^33^. *F. saccharivorans* is a Gram-positive, obligately anaerobic, non-motile, non-spore-forming, spindle-shaped bacterium isolated from human faeces^41^. All the bacteria sequenced in this study, except *H. canadensis*, have not previously been reported in pigs.

The microbiota in post-weaning piglets formed a more coherent cluster with greater similarity indices than that in the pre-weaning piglets. Weaning causes significant changes in the mucosa-associated bacterial communities and digesta samples from the jejuna of piglets^34^. The clustering analyses performed in this study indicated that post-weaning piglets formed a coherent cluster, with similarity indices above 69%. Interestingly, the rpBD2-supplemented mucosal microbiota showed high sequence similarities (>80%), whereas the rpBD2-supplemented digesta microbiota showed the lowest coherence. However, the Shannon index of diversity for the mucosal samples from the PC group was almost the same as that for the 5PD-treated group. These phenomena suggest that rpBD2 increased the coherence of the mucosal microbiota among different pens of piglets but did not affect the microbial diversity on the intestinal mucosa.

Before the establishment of a developed immune system, which generally takes 4–5 weeks, newly weaned piglets are very susceptible to diseases and stressors. At this stage, dietary supplementation with innate immunity factors is important for the growth performance and health of the weaning piglets^28^. In this study, we have demonstrated that dietary supplementation with crude rpBD2 has beneficial effects on the growth performance and intestinal morphology of weaned piglets, reducing the incidence of PWD and the numbers of potential pathogens in the caecum. Therefore, rpBD2 can potentially be used as an alternative to traditional antibiotic feed additives for weaned piglets on commercial farms. The detailed mechanism(s) by which rpBD2 promotes the growth performance of weaned piglets and improves their intestinal health requires further clarification.

## Methods

### Preparation of crude rpBD2

The method for preparing crude rpBD2 has been described by Peng *et al.*^19^. Briefly, the rpBD2 supernatant was harvested from the culture medium of rpBD2-experssing *P. pastoris* X-33, dialyzed with a 1-kDa nanofiltration membrane (Laungy Membrane Filtration Technology, Shanghai, China), and spray-dried. The resulting crude rpBD2 powder was confirmed with an inhibition zone assay^42^ against *S. aureus* ATCC 6538 and with tricine–SDS–PAGE^43^. The concentration of rpBD2 in the crude powder was quantified with a Porcine β Defensin 2 Enzyme-linked Immunosorbent Assay (ELISA) Kit (Cloud-Clone, Houston, TX, USA). The crude powdered *P. pastoris* X-33 culture supernatant was used as the blank control.

### Weaned piglets and dietary treatments

All animal experiments were performed in accordance with protocols approved by the Ethics Committee of the State Key Laboratory of Direct-Fed Microbial Engineering, China National Center for Food Safety Risk Assessment and the Beijing Institute of Microbiology and Epidemiology. In total, 120 weaned piglets (Landrace × Yorkshire × Duroc; 9.375 ± 0.017 kg in body weight [BW]; 21 ± 2 days old; 60 males and 60 females) with the same ancestry were used in a 28-day growth study on a commercial farm in Henan Province in July 2014. The piglets were randomly assigned to four groups for dietary treatment: PC, 1PD, 5PD, and 15PD. The ingredients and chemical composition of the basal diet are shown in Table 4. The groups were composed of three replicate pens with 10 piglets each, and all groups were supplied with either antibiotics or rpBD2. The experiment was divided into phases I (0–14 days post-weaning) and II (14–28 days post-weaning).

The diets were formulated to meet or exceed the National Research Council guidelines^44^ for 10–20-kg pigs. The pigs were housed in temperature-controlled (29 °C) nursery rooms and grouped in elevated pens with wire flooring. Feed and water were available to the pigs *ad libitum*. The pigs were individually weighed on an empty stomach on days 0, 14, and 28. Feed consumption was recorded on days 14 and 28. All feed remaining in the feed trough at the next feeding period was weighed and subtracted from the daily allowance to determine the actual daily feed intake. Growth performance indicators, such as BW, ADG, ADFI, and G/F, were determined for each pen at the end of every phase.

### Faecal consistency and the incidence of PWD

The occurrence of PWD in each piglet was assessed visually each afternoon using the method of Hart and Dobb^45^. The scores were: 0 = normal, firm faeces; 1 = possible slight diarrhoea; 2 = definitely unformed/moderately fluid faeces; and 3 = very watery and frothy diarrhoea. A cumulative diarrhoea score per diet and day was then calculated^25^. The occurrence of diarrhoea was defined as the maintenance of faecal scores of 2 or 3 for 2 consecutive days and was determined with the formula: diarrhoea incidence (%) = ([number of piglets with diarrhoea within a treatment]/[number of piglets × total experimental days]) × 100, where ‘number of piglets with diarrhoea’ was the total number of piglets with diarrhoea observed each day^46^.

### Small intestinal morphology

To study the effects of rpBD2 on the small intestinal morphology, six pigs were randomly selected from each treatment group (two piglets per pen, one male and one female, reflecting the average BW of the pen) and killed by electrocution on day 28. The samples of intestinal segments from the middle regions of the duodenum, jejunum, and ileum, after removal of their contents, were aseptically isolated, flushed with physiological saline, and submerged in a fixative solution (0.1 M collidine buffer, pH 7.3) containing 3% glutaraldehyde, 2% paraformaldehyde, and 1.5% acrolein, for further analysis. Three cross-sections were prepared from each intestinal sample after staining with haematoxylin and eosin, using standard paraffin-embedding and staining procedures^33^. In total, 10 intact, well-oriented crypt–villus units were selected in triplicate for each intestinal cross-section (30 measurements of each sample; a total of 180 measurements per dietary treatment). The villus heights and crypt depths were determined with an image processing and analysis system (Leica Imaging Systems, version 1, Cambridge, UK)^23^.

### Intestinal microfloral analysis

Fresh digesta from cecal samples (approximate middle segments) from piglets in the PC and 5PD treatment groups were collected aseptically, immediately immersed in liquid nitrogen, and stored at −20 °C. To analyse the microflora intimately attached to the cecal mucosa, the luminal fluid was drained, and the middle segments of the caecum (3–4 cm) were excised, washed with sterile phosphate-buffered saline (pH 7.0), immediately snap frozen in liquid nitrogen, and stored at −20 °C.

For the DGGE analysis, total DNA was extracted from the digesta and mucosal samples with a TIANamp Stool DNA Kit (Tiangen, Beijing, China). The variable V3 region of 16S rRNA was amplified with PCR using the universal primers 341F (5′-CCTACGGGAGGCAGCAG-3′) and 534R (5′-ATTACCGCGGCTGCTGG-3′), and a GC clamp (5′-CGCCCGCCGCGCGCGGCGGGCGGGGCGGGGGCACGGGGGG-3′) was attached to the 5′-terminus of 341F^47^,^48^. The PCR products were loaded onto 8% polyacrylamide gels containing 37.5:1 acrylamide:bisacrylamide and a denaturing gradient of 38–51% (100% denaturant was equivalent to 7 M urea and 40% deionised formamide) using the DCode Universal Mutation Detection System (Bio-Rad Laboratories, Hercules, CA, USA). Electrophoresis was initiated by pre-running the samples for 10 min at 200 V and then continued at a fixed voltage of 85 V for 12 h at 60 °C^49^. The gels were stained with Gene Green I (Tiangen) after electrophoresis and scanned with the Quantity One software (version 4.6.3, Bio-Rad, USA). The DGGE profiles were clustered using the unweighted pair group method with arithmetic averages (UPGMA) in the MEGA 4.0 software. Preliminary diversity data were collected from the DGGE bands with the Quantity One analysis software, and Shannon’s diversity index was calculated to measure microfloral diversity^49^.

Six 16S rRNA PCR products from prominent bands that differed between the cecal digesta and mucosal samples from the PC and 5PD groups were selected, purified with the Gel Recovery Purification Kit (Tiangen), cloned into the pMD18-T vector (Takara, Dalian, China), and used to transform *E. coli* DH5α cells. Ten clones were randomly selected for each band and the cloned inserts amplified with PCR. Sequencing was performed by Invitrogen (Beijing, China) using a universal primer pair for the pMD18-T vector: M13F (−40) 5′-GTTTTCCCAGTCACGAC-3′ and M13R (−26) 5′-CAGGAAACAGCTATGAC-3′. The 16S rRNA gene sequences are available in GenBank under accession numbers KM220765, KM220766, KM220767, KM220768, KM220769, and KM220770.

### Statistical analysis

The data were analysed according to a randomized complete block design using the general linear model procedure of the SAS Statistical Software (SAS Institute, Cary, NC, USA). One-way analysis of variance (ANOVA) was applied to all parameters. When significant differences were observed among treatment means, they were separated with Tukey’s honest significant difference test. ‘Pen’ was the experimental unit for the analysis of all parameters. Differences with *P* < 0.01, <0.05, and <0.10 were considered extremely significant, significant, and a trend, respectively.

## Additional Information

**How to cite this article**: Peng, Z. *et al.* Use of recombinant porcine β-defensin 2 as a medicated feed additive for weaned piglets. *Sci. Rep.* **6**, 26790; doi: 10.1038/srep26790 (2016).

## Supplementary Material

Supplementary Fig. S1

## Figures and Tables

**Table 1:** Growth performance and incidence of diarrhoea in weaned piglets.

Item	PC	PD			SEM	*P* Value
		1	5	15		
Body weight, kg						
Initial weight (1 d)	9.38^a^	9.35^a^	9.38^a^	9.39^a^	0.033	0.984
Final weight (28 d)	16.79^b^	17.04^b^	17.62^a^	16.98^b^	0.106	0.007
Average daily weight gain, g/d						
1–14 d	159.5^a^	167.9^a^	173.8^a^	164.2^a^	3.020	0.442
14–28 d	369.5^b^	381.7^b^	414.3^a^	378.1^b^	5.806	0.006
1–28 d	264.5^b^	274.8^b^	294.0^a^	271.1^b^	3.754	0.005
Average daily feed intake, g						
1–14 d	333.6^ab^	317.1^bc^	344.0^a^	309.3^c^	4.977	0.022
14–28 d	625.0^b^	634.5^b^	717.9^a^	640.3^b^	12.469	0.004
1–28 d	479.3^b^	475.8^b^	531.0^a^	474.8^b^	7.575	0.001
Feed conversion (g feed/g weight gain)						
1–14 d	2.10^a^	1.89^a^	1.98^a^	1.89^a^	0.047	0.409
14–28 d	1.69^a^	1.66^a^	1.73^a^	1.69^a^	0.012	0.211
1–28 d	1.81^a^	1.73^b^	1.80^a^	1.75^b^	0.012	0.008
Diarrhoea incidence (%)	4.4^a^	3.9^ab^	2.4^c^	2.9^bc^	0.277	0.009

PC: positive control; PD: rpBD2 treatment groups; SEM: standard error of the mean. ^a,b,c^Mean values within a row with unlike superscript letters are significantly different (*P* < 0.05).

**Table 2:** Characteristics of the duodenum, jejunum, and ileum in weaned piglets (28 days).

Item	PC	PD			*P*Value
		1	5	15	
Duodenum					
Villus height, μm	541.1 ± 66.3^b^	500.8 ± 65.7^b^	702.3 ± 55.9^a^	656.2 ± 61.1^a^	0.025
Crypt depth, μm	351.7 ± 54.4^a^	296.6 ± 65.2^a^	317.6 ± 32.5^a^	361.1 ± 50.7^a^	0.443
Villus height/crypt depth	1.5 ± 0.5^a^	1.8 ± 0.6^a^	2.2 ± 0.3^a^	1.8 ± 0.4^a^	0.362
Jejunum					
Villus height, μm	507.7 ± 60.4^b^	540.7 ± 36.9^b^	651.4 ± 20.5^a^	623.3 ± 22.2^a^	<0.010
Crypt depth, μm	211.8 ± 37.0^b^	297.7 ± 23.4^a^	252.0 ± 19.0^ab^	303.6 ± 61.7^a^	0.063
Villus height/crypt depth	2.5 ± 0.8^a^	1.8 ± 0.3^a^	2.6 ± 0.3^a^	2.1 ± 0.5^a^	0.307
Ileum					
Villus height, μm	369.0 ± 66.1^ab^	454.1 ± 68.9^a^	327.6 ± 44.2^b^	384.3 ± 34.6^ab^	0.110
Crypt depth, μm	243.3 ± 29.4^ab^	272.7 ± 55.2^a^	200.7 ± 21.5^b^	312.0 ± 32.0^a^	0.031
Villus height/crypt depth	1.5 ± 0.5^a^	1.7 ± 0.6^a^	1.7 ± 0.2^a^	1.2 ± 0.02^a^	0.461

PC: positive control; PD: rpBD2 treatment groups; SEM: standard error of the mean. ^a,b,c^Mean values within a row with unlike superscript letters are significantly different (*P* < 0.05).

**Table 3:** Alignment of sequenced clones to the most similar GenBank sequences.

Sequenced clones	Corresponding sample	Length of 16S rDNA V3 fragments (bp)	GenBank accession number	Most similar GenBank sequence			
				Aligned site	% similarity	Bacterium	Accession number
1	CM1	196	KM220765	334–524	87	*Solobacterium moorei*	NR_113039.1
2	CD2	171	KM220766	296–466	100	*Helicobacter canadensis*	NR_115104.1
3	CM1	171	KM220767	349–519	100	*Eubacterium eligens*	NR_074613.1
4	CM1	171	KM220768	327–497	100	*Coprococcus comes*	NR_044048.1
5	CM2	173	KM220769	346–516	99	*Clostridium polysaccharolyticum*	NR_119085.1
6	CM5	171	KM220770	333–503	100	*Fusicatenibacter saccharivorans*	NR_114326.1

**Table 4:** Ingredients and chemical composition of the basal diet.

Ingredients	%	Nutrient composition	%
Maize	59.37	Crude protein	19.19
Soybean meal	25.00	Calcium	0.583
Fishmeal	4.00	P	0.464
Dried whey power	4.00	Lysine	1.198
Cream from bovine milk	5.00	Methionine	0.397
Limestone	0.30	Threonine	0.850
Monocalcium phosphate	1.10	Digestible energy†/kg feed	14.3 MJ
Anti-mould agent	0.10	NA	NA
Antioxidant	0.02	NA	NA
Vitamin premix*	0.04	NA	NA
Trace mineral premix#	0.30	NA	NA
Salt	0.30	NA	NA
Flavour	0.06	NA	NA
L-lysine-HCl	0.23	NA	NA
L-methionine	0.05	NA	NA
L-threonine	0.05	NA	NA

^*^Vitamin premix consisted of the following ingredients per kilogram of complete diet: 11,000 IU (3,300 μg) vitamin A, 1,100 IU (27.5 μg) vitamin D3, 22 IU (14.67 μg) vitamin E, 4 mg menadione as dimethylpyrimidinol bisulfite, 0.03 mg vitamin B12, 28 mg d-pantothenic acid, 33 mg niacin, and 0.08% choline chloride.

^#^Trace mineral premix consisted of the following ingredients per kilogram of complete diet: 165 mg Zn (ZnSO_4_), 165 mg Fe (FeSO_4_), 33 mg Mn (MnSO_4_), 16.5 mg Cu (CuSO_4_), 297 μg I (CaI_2_), and 297 μg Se (Na_2_SeO_3_);

^†^Digestible energy was based on calculated values. NA: not applicable.
