# Supplementary material for: Use of recombinant porcine β-defensin 2 as a medicated feed additive for weaned piglets
Source: Sci Rep. 2016 May 26;6:26790. doi: 10.1038/srep26790 (PMC4880912; doi:10.1038/srep26790)
Supplement: Supplementary Fig. S1 [file srep26790-s1.pdf]

Use of recombinant porcine β-defensin 2 as a medicated feed additive for weaned piglets

Zixin Peng[1,3], Anru Wang[3], Linqi Xie[3], Weiping Song[3], Jie Wang[2], Zhe Yin[2], Dongsheng Zhou[2,]* & Fengqin Li[1,]*

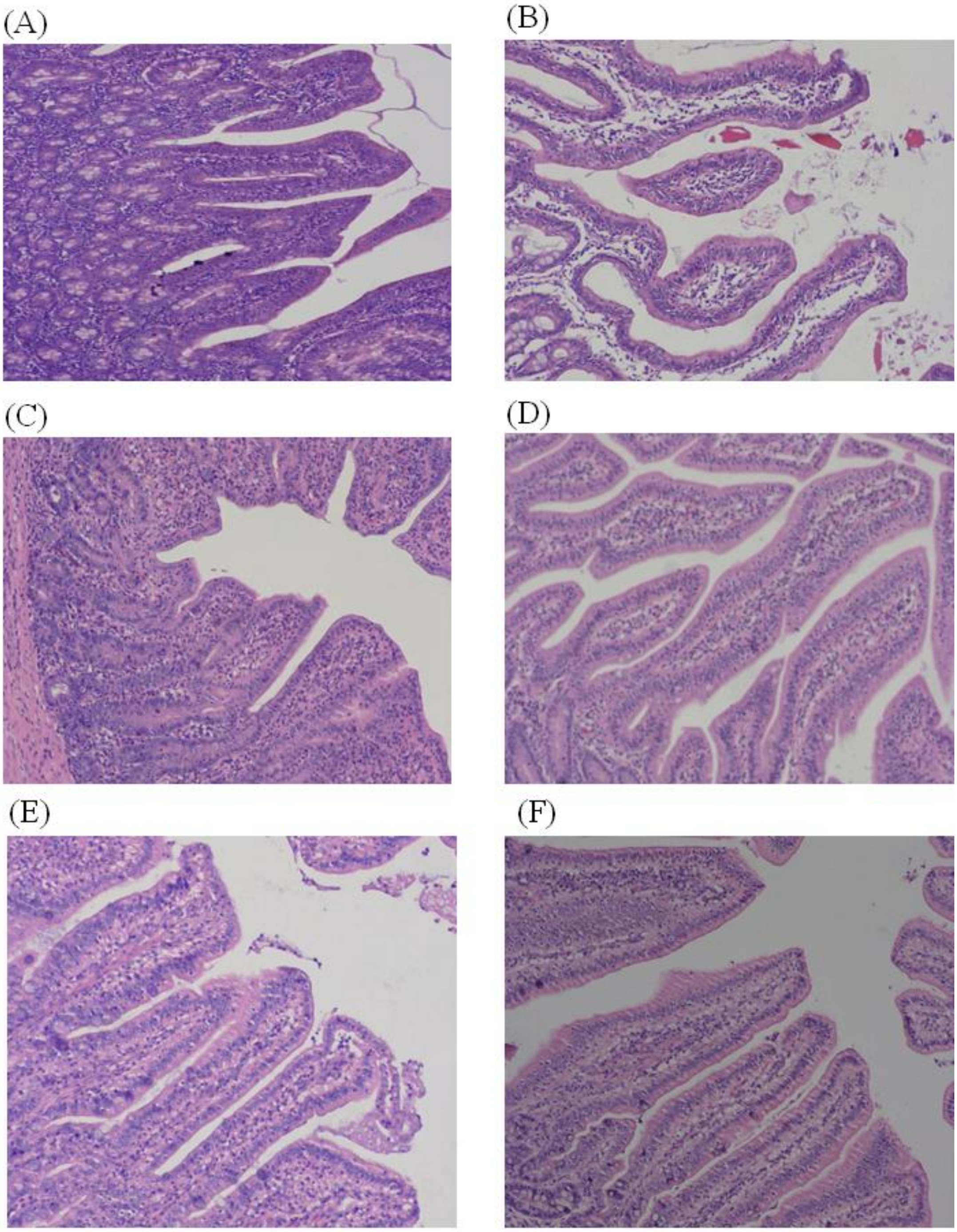

Supplementary Fig S1. Rrepresentative micrographs of small intestinal morphology in weaned piglets (28 days). (A) and (B) are duodenum morphology of PC and 5PD, respectively; (C) and (D) are jejunum morphology of PC and 5PD, respectively; (E) and (F) are ileum morphology of PC and 5PD, respectively.
